# Results of Standard Stapler Closure of Pancreatic Remnant After Distal Spleno-Pancreatectomy for Adenocarcinoma

**DOI:** 10.3389/fsurg.2020.596580

**Published:** 2020-11-05

**Authors:** Giulio Illuminati, Saverio Cerasari, Rocco Pasqua, Priscilla Nardi, Chiara Fratini, Sébastien Frey, Antonio Iannelli, Pierluigi Marini

**Affiliations:** ^1^The Department of Surgical Sciences, The University of Rome “La Sapienza”, Rome, Italy; ^2^The Department of Digestive Surgery and Liver Transplantation Unit, University Hospital of Nice Archet, University of Cote d'Azur, Nice, France; ^3^The Department of General and Emergency Surgery, San Camillo–Forlanini Regional Hospital, Rome, Italy

**Keywords:** distal pancreatectomy, stapler closure, pancreatic fistula, adenocarcinoma, pancreatic remnant

## Abstract

**Background/Aim:** The purpose of this study was to evaluate the results of stapled closure of the pancreatic remnant after cold-knife section of the pancreatic isthmus and distal pancreatectomy for adenocarcinoma.

**Methods:** A retrospective evaluation of 57 consecutive patients undergoing distal spleno-pancreatectomy for adenocarcinoma was performed. The pancreatic isthmus was systematically straight-sectioned with a cold knife, and the remnant was stapled close without additional stitches or adjuncts. The study's main endpoints were postoperative mortality, the occurrence of a pancreatic fistula, the need for a re-operation, the postoperative length of stay in the hospital, the rate of re-admission, and late survival.

**Results:** Postoperative mortality was absent. Seventeen patients (29.8%) presented a pancreatic fistula of grade A in seven cases (41.2%), grade B in eight cases (47.1%), and grade C in two cases (11.8%). Re-operation was required in the two patients (3.5%) with grade C fistula in order to drain an intra-abdominal abscess. The mean postoperative length of stay in the hospital was 15 days (range, 6–62 days). No patient required re-admission. Twenty-nine patients (50.8%) were alive and free from disease, respectively, 12 patients (21.1%) at 12 months, 13 patients (22.8%) at 60 months, and four patients (7.0%) at 120 months from the operation. The remaining patients died of metastatic disease 9–37 months from the operation. Lastly, disease-related mortality was 49.1%.

**Conclusion:** Stapler closure of the pancreatic remnant allows good postoperative results, limiting the formation of pancreatic fistula to the lower limit of its overall reported incidence.

## Introduction

The incidence of postoperative pancreatic fistula (POPF) after distal pancreatectomy varies from 10 to 40% (1–11). POPF is caused by leakage from the pancreatic duct branches that connect the main pancreatic duct with the cut surface (12). Although results of pancreatic surgery significantly improved in the last decades, the incidence of POPF has not significantly decreased, and it remains a cause of significant postoperative morbidity (13) as it may be followed by complications leading to re-operation (14), prolonged hospital stay, and increased costs (15).

Several methods of pancreatic transection and stump closure techniques have been proposed in order to reduce the incidence of POPF (10), including scalpel section and suture (16), ultrasound section (17), stapler closure (18), stapler and glue (19), Roux-en-Y drainage of the stump (20), ligasure section (21), and patch closure of the stump (22). However, so far, no conclusive evidence of superiority of a technique over the others is really available, and a recent study concluded that the rate of POPF after distal spleno-pancreatectomy (DP) is probably independent of the technique of pancreatic closure (13).

Such variability in results and rate of fistula formation may be related both to technical factors and to patient-related variables, including American Society of Anesthesiologists (ASA) score, body mass index (BMI)/overweight, diabetes, hypoalbuminemia, pancreatic thickness, indication for operation (invasive ductal carcinoma, pancreatitis, neuroendocrine tumors), level of pancreatic transection, length, and complexity of operation, associated splenectomy, and intra-operative bleeding (12, 23–26).

The purpose of this study was, therefore, to retrospectively evaluate the results of stapler closure of the pancreatic remnant, with straight cold-knife section of the distal pancreas, when performing distal pancreatectomy for adenocarcinoma.

## Patients and Methods

From January 2000 to December 2019, patients undergoing distal pancreatectomy at two academic, tertiary care hospitals and one regional tertiary care center were prospectively entered into a database and were retrospectively reviewed. For the study's purposes only, the records of patients undergoing DP for an adenocarcinoma arising from the body–tail of an otherwise normal pancreas were retained. Patients undergoing distal pancreatectomy for other tumors and diseases or from adenocarcinomas arising on the setting of a chronic pancreatitis were excluded from the study. Patients with metastatic disease were also excluded from the study. Fifty-seven (57) patients met the study's criteria. Informed consent for operation was obtained from all the patients, whereas approval from the Institutional Ethics Committee was waived due to the retrospective nature of the study.

### Surgical Technique

**Figure 1:**
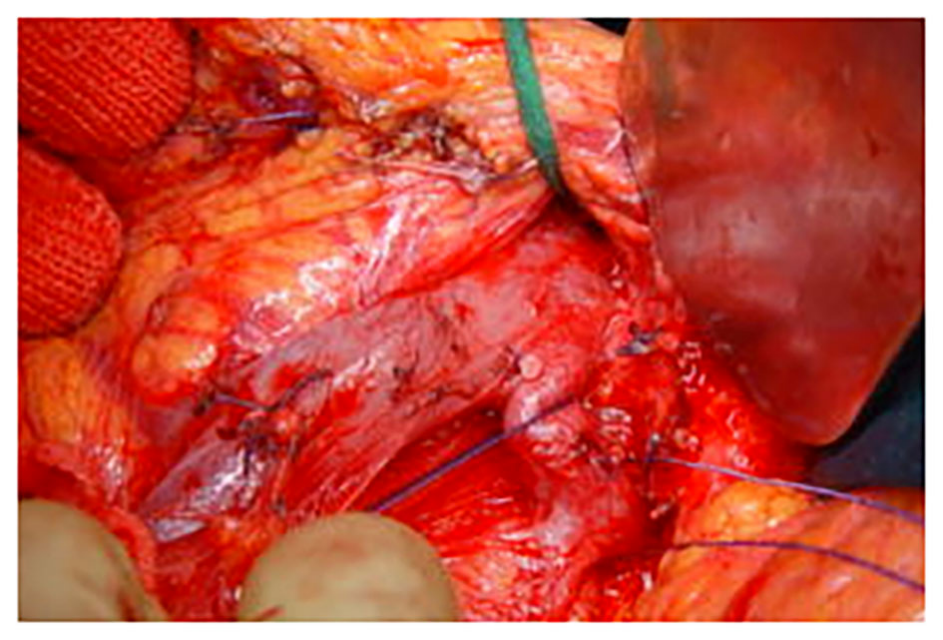
Intraoperative view. The spleno-mesenteric confluence and the splenic and the inferior mesenteric veins are separately isolated, ligated, and sectioned.

**Figure 2:**
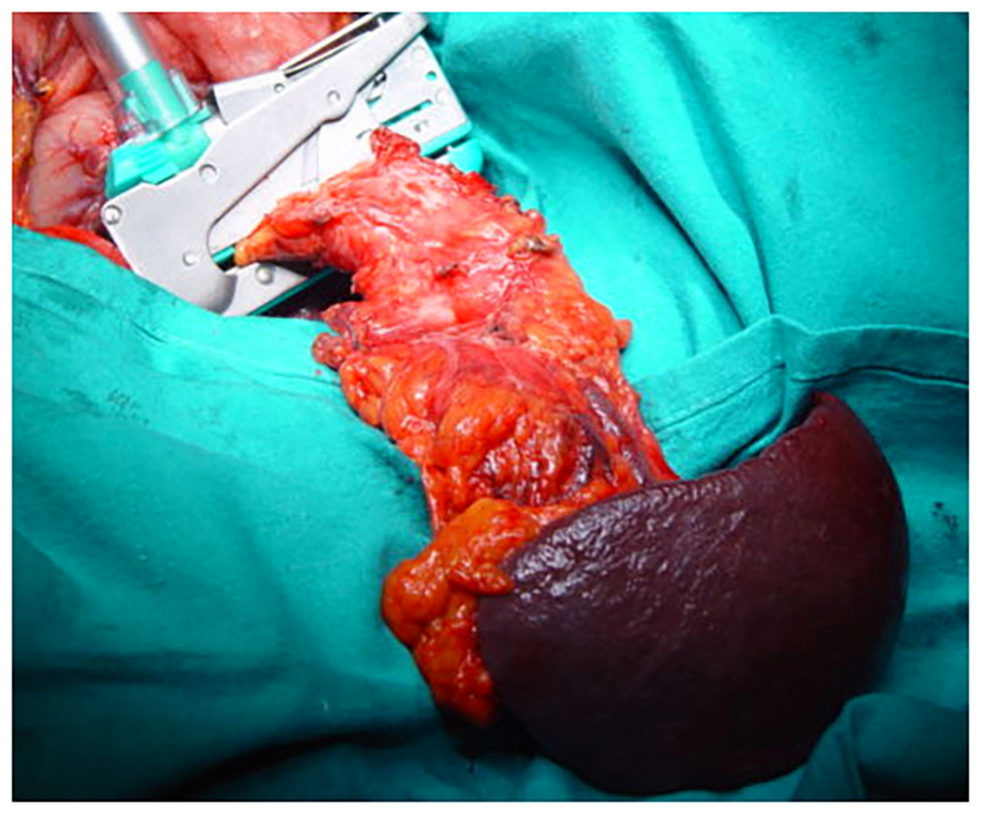
Intraoperative view. A linear stapler (TA 90−4.8 mm) is applied on the pancreatic isthmus at the level of the antero-left side of the portal vein and fired.

**Figure 3:**
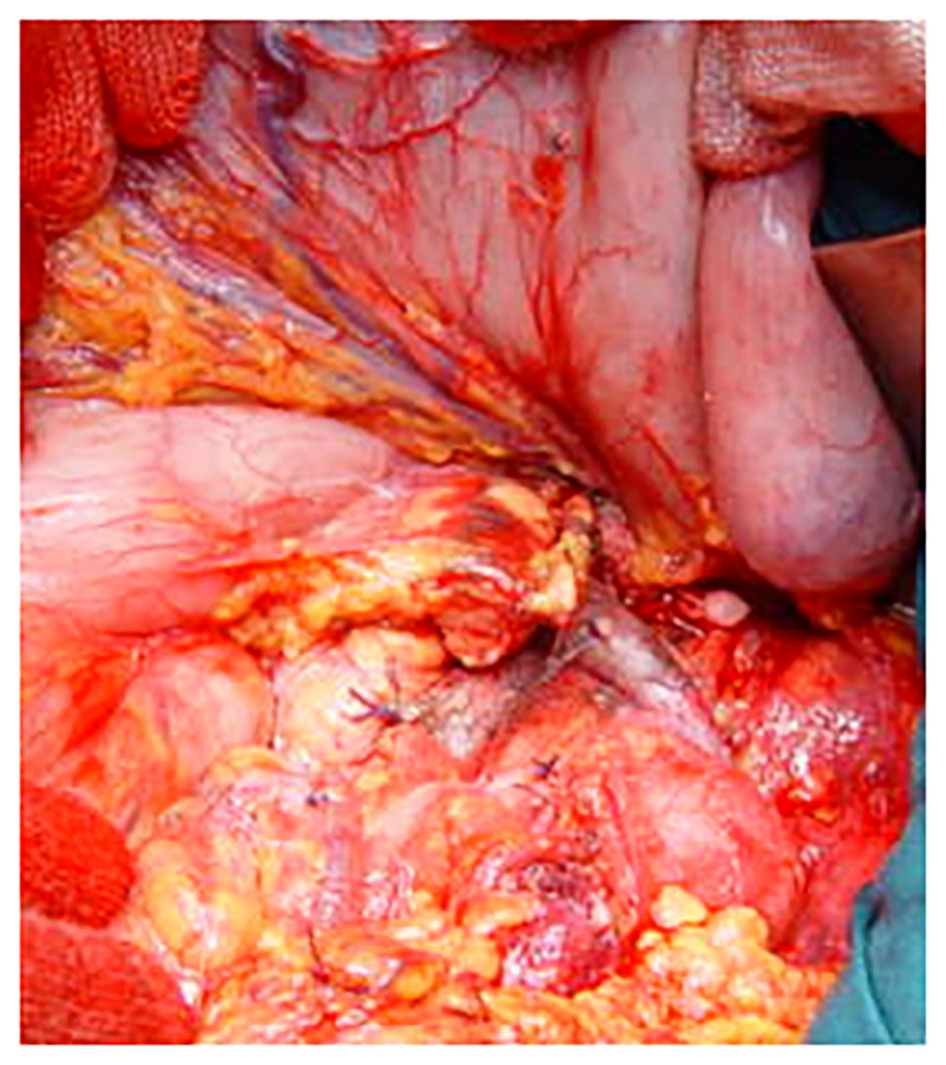
Intraoperative view. No additional suture is applied on the pancreatic remnant after simple stapler suture and distal splenopancreatectomy have been completed.

The technique of DP was standard and superposable in the three centers participating in the study, all participating to the same program of residents' training and surgeons' exchange, with the three senior surgeons performing the operations. Access was gained through a bi-subcostal incision. The splenic artery was controlled, ligated, and sectioned at its origin. The superior mesenteric vein and the portal vein were controlled caudally and cranially at the isthmus of the pancreas progressively freed on its dorsal aspect. The spleno-mesenteric confluence and the splenic and inferior mesenteric vein were separately ligated and sectioned (Figure 1). A linear stapler (TA 90-−4.8 mm or Endo GIA 60 mm, Autosuture Covidien Medtronic, Minneapolis, MN, USA) was applied on the pancreatic isthmus at the level of the antero-left side of the portal vein and fired (Figure 2). The pancreas was straight-sectioned on the left border of the stapler with a cold knife. Sectioning of the pancreas with a cold knife was not necessary when using the Endo GIA device, and a normal stapler-based transection was performed. Spleno-pancreatectomy was performed *en bloc* with lymphatic tissue, and the surgical specimen was sent for pathological examination. Hemostasis of the cut surface on the pancreatic remnant was performed, if needed, with separate stitches of 6-0 polypropylene monofilament. No additional hand-sewn suture of the pancreatic stump was performed (Figure 3). Two 28-mm silicone drains were left in place: one facing the pancreatic stump and one in the left sub-diaphragmatic space, exiting from the left side of the abdomen (10). Postoperatively, the drains were checked and squeezed at least twice a day to prevent their obstruction. The amylase content on the drained fluids was measured on postoperative days 1, 3, and 5 and then daily in case of persistent output. A control CT scan was systematically performed on postoperative days 5–7 and, if normal, was followed by drain removal. Systematic postoperative administration of somatostatin analogs in an uneventful postoperative course was not performed.

### Outcome Definition and Measurement

As the main study's outcomes, postoperative mortality, the occurrence of a pancreatic fistula, the need for a re-operation, postoperative length of stay in the hospital, the rate of re-admission, and late survival were considered.

Postoperative mortality was defined as any death occurring within 30 days from the operation or during the whole postoperative stay in the hospital. POPF was defined as an abnormal communication between the pancreatic ductal system and another epithelial surface containing pancreatic fluid (27) and was diagnosed according to the criteria defined by the ISGPS (28) as a measurable drain fluid output, between postoperative days 3 and 7, containing an amylase concentration that was 3-fold that of normal serum laboratory values (9, 28). The clinical entity of POPF was classified as grade A if consisting only in a biochemical leakage with no clinical impact, not requiring any further treatment in addition to drains placed at operation, grade B if associated with an abdominal fluid collection requiring further drainage through an interventional radiology procedure, and grade C if requiring re-operation as for abscess formation or hemorrhage.

Postoperative length of stay was defined as the number of days between postoperative day 1 and the day of discharge from the hospital. Re-admission was defined as any new hospitalization related to the operation occurring after discharge from the hospital. Late survival was defined as the overall survival minus any disease-related death during follow-up, whose mean length was 16 months (range, 9–48 months). Late disease-related mortality is mortality due to disease progression occurring after a potentially curative resection and excluding postoperative deaths.

## Results

Of the 57 enrolled patients, 29 were male with a mean age of 64 years (range, 36–81 years). The mean BMI (kg/cm^2^) of the patients was 24.5 (range, 18–38). Fourteen patients (24.6%) were ASA score I, 34 patients (59.6%) were ASA score II, seven patients (12.3%) were ASA score III, and two patients (3.5%) were ASA score IV. Twelve patients (21.1%) were diabetic, and eight patients (14.0%) underwent neo-adjuvant chemo/radiotherapy before the operation. The mean length of operation was 244.5 min (range, 125–380 min). The mean intra-operative blood loss was 200 ml (range, 50–2,100 ml), and five patients (8.8%) required blood transfusions. All the patients presented a clearance margin of at least 1.5 cm from the tumor at pathological examination (Table 1).

**Table 1:** Demographics and main clinical variables of the patients' population.

**Variable**	**Measure**
Age (years)	64 (range, 36–81)
Male (*N*, %)	29, 50.9%
BMI (kg/cm^2^)	24.5 (range, 18–38)
ASA score I (*N*, %)	14, 24.6%
ASA score II (*N*, %)	34, 59.6%
ASA score III (*N*, %)	7, 12.3%
ASA score IV (*N*, %)	2, 3.5%
Mean length of operation (minutes)	244.5 (range, 125–380)
Intraoperative blood loss (ml)	200 (range, 50–2,100)
Patient requiring blood transfusion (N)	5, 8.8%

*BMI, Body Mass Index; ASA, American Society of Anesthesiologists*.

### Main Outcomes

No patient died in the postoperative period in this series.

Seventeen patients (29.8%) presented a POPF of grade A in seven cases (41.2%), grade B in eight cases (47.1%), and grade C in two cases (11.8%). A grade B fistula was treated with interventional drainage in six patients. In no case of grade B fistula was intensive care unit transfer or any major systemic complication observed. Re-operation was required in two patients (3.5%) with grade C fistula in order to drain an intra-abdominal abscess: re-operation required a precautionary stay in intensive care unit for 48 and 72 h, respectively, without any major systemic complication. The mean postoperative length of stay in the hospital was 15 days (range, 6–62 days). No patient required re-admission for operation-related events after discharge from the hospital.

**Figure 4:**
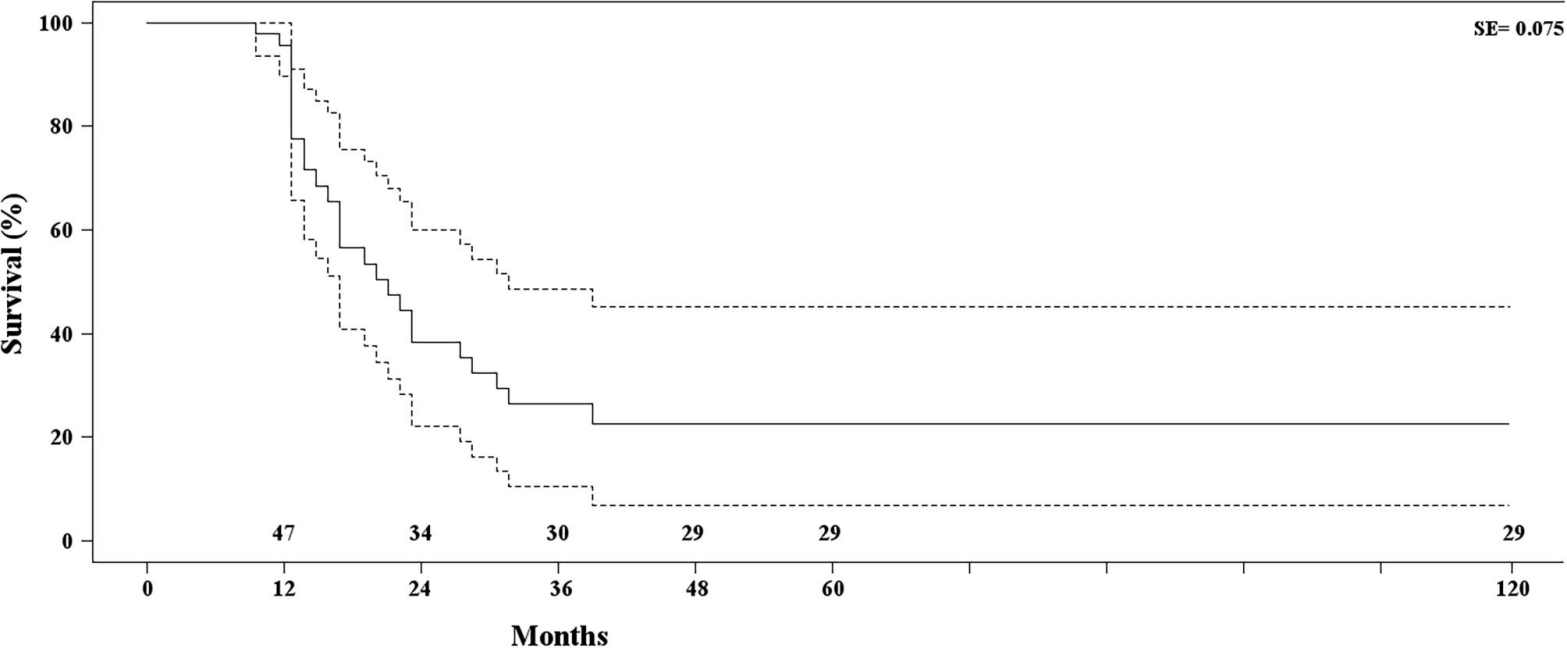
Kaplan–Meier estimate of patients' survival. Dotted lines define standard error.

Twenty-nine patients (50.8%) were alive and free from disease, respectively, 12 patients (21.1%) at 12 months, 13 patients (22.8%) at 60 months, and four patients (7.0%) at 120 months from the operation. The remaining patients died of metastatic disease 9–37 months from the operation. Late disease-related mortality was 49.1%; survival was assessed with life-table analysis and outlined with Kaplan–Meier curves (Table 2, Figure 4).

**Table 2:** Main study's outcomes.

**Variable**	**Measure**
Postoperative mortality (*N*, %)	0
Postoperative pancreatic fistula (*N*, %)	17, 29.8%
Grade A (*N*, %)	7, 41.2%
Grade B (*N*, %)	8, 47.1%
Grade C (*N*, %)	2, 11.8%
Mean postoperative length of stay in the hospital (days)	15 (range, 6–62)
Patients readmitted to the hospital (*N*, %)	0
Late disease related mortality	49.1%

*Mean length of follow up: 28 months (range, 9–120 months)*.

## Discussion

The results of this study show that straight, cold-knife section of the pancreatic isthmus associated with simple staple suture of the pancreatic remnant allows satisfactory postoperative results, in terms of POPF formation, when performing a DP for adenocarcinoma. These results are obtained with a simple and standard technique, scarcely modified by variables related to the single operating surgeon and particularly valuable for laparoscopic resections which are gaining increasing popularity. It can be assumed that, with the gain of popularity of laparoscopic distal pancreatectomy, stapler closure of the pancreatic remnant will be performed with increasing frequency. Several studies, including also randomized trials, tested different techniques of closure of the pancreatic remnant after DP, without conclusively demonstrating the superiority of any technique over the others (29). As a support to this statement, no difference in POPF formation has been detected in a large trial between staple and hand-sewn closure of the pancreatic stump and between systematic administration and no administration of somatostatin analogs in the postoperative period (9). Besides a recent metaanalysis and randomized trial observing a trend toward a possible advantage of round ligament and seromuscular patch closure of the pancreatic stump in preventing POPF (8, 22), another randomized trial demonstrated that neither the addition of falciform ligament patch nor the application of fibrin glue reduced the rate or the severity of POPF after hand-sewn or stapler closure of the pancreatic stump (29). For a study observing a higher incidence of POPF formation after stapler closure (6), other reports have shown that the technique itself does not influence the incidence of POPF (30–32).

The incidence of POPF in this study is within the lower limit of its overall incidence reported in the literature, and this study's results are comparable to those of a large retrospective analysis supporting the superiority of stapler closure over standard suture techniques (25, 33) (Table 3).

**Table 3:** Outcomes according to closure of pancreatic stump.

**References**	**Study group (*n*)**	**Postoperative pancreatic fistula (%)**	**Mortality (%)**
Diener et al. (9)	Stapler (177) vs. suture (36)	36 vs. 37	0 vs. 1
Ban et al. (25)	Stapler (177) vs. suture (36)	21 vs. 50.6	0 vs. 0
Kawai et al. (33)	Hand sewn closure (32) vs. stapler closure (45)	18.7 vs. 11.1	0 vs. 0
Kleff et al. (6)	Stapler (145) vs. suture (97)	15.9 vs. 9.3	2.8 vs. 2.1
Pannegeon et al. (5)	Stapled (108) vs. hand sewn (67)	28.5 vs. 31.4	0 vs. 0
Chikhladze et al. (13)	Hand sewn with interrupted U-suture (201) and stapler closure (52)	69 vs. 57	0 vs. 0
Sledzianowski et al. (4)	Stapled (52) sutured (43)	6 vs. 15	0 vs. 2
Probst et al. (34)	Stapled (1,000) hand sewn (1,000)	35 vs. 36.3	0.6 vs. 1.1
Present series	Stapled (57)	29.8	0

Stapler closure of the pancreatic stump may be supported by some experimental evidence based on the physiopathology of the pancreatic fistula (12). POPF is caused by leakage from regenerated pancreatic duct branches, whenever flow disturbances evolving toward necrosis occur over the cut surface of the pancreas. Hand-sewn stump closure may induce a higher burst of pressure and more blood flow disturbances with tissue necrosis than staple closure. A higher extension of tissue necrosis with hand-sewn suture compared with stapler suture has been experimentally observed at a pathology examination in the pancreatic stump of mongrel dogs (12). Normal pancreatic parenchyma is soft and fragile (35), and a tight hand-sewn ligation may reduce blood flow to the pancreatic stump, thus leading to ischemia, necrosis, and development of POPF (12). Blood flow discrepancies may alter the proportion between regenerated pancreatic ducts under tension and granulation tissue with fluid from regenerated ducts flowing through the necrotic tissue. Compared to the tension of hand-sewn closure, stapler closure is less strong and tight, thus reducing the probability of regenerated tissue necrosis and fistula formation (12). In addition, hand-sewn closure is more dependent on the skills of individual surgeons and the stitch technique used, whereas staple suture allows a more standardized closure performance (34). Compared to harmonic scalpel and other electric/electronic devices, cold-knife section of the pancreas does not induce any potential termic lesion of the pancreatic remnant close to the section surface, thus contributing to a lower incidence of tissue necrosis and consequent fluid spillage.

### Strengths and Limitations

The strengths of this study consist of the homogeneous sample of patients, overall comparable treatment, and standardized surgical technique. The major limitations are its retrospective nature and long time-span. The long time-span also explains the overall quite long hospital stay: it was customary, in the early years of the study, to do most of preoperative study during hospitalization and to prolong postoperative stay even in case of an uneventful postoperative course. Nonetheless, patients' data were carefully assessed, and the results were objectively assessed, with single patient's variables possibly hindering surgical outcomes reduced at minimum.

### Conclusion

In conclusion, the results of this study show that stapler closure of the pancreatic remnant and straight section of the pancreas with cold knife, when performing DP for adenocarcinoma, allows good postoperative results, limiting the formation of POPF to the lower rate of its overall reported incidence.

## Data Availability Statement

The data analyzed in this study is subject to the following licenses/restrictions: privacy rules. Requests to access these datasets should be directed to giulio.illuminati@uniroma1.it.

## Ethics Statement

Ethical review and approval was not required for the study on human participants in accordance with the local legislation and institutional requirements. The patients/participants provided their written informed consent to participate in this study.

## Author Contributions

GI, AI, and PM: conception, design, and critical revision of the article. SC, RP, PN, CF, and SF: data collection. GI, SC, AI, and PM: analysis and interpretation. GI and SC: writing the article. GI, SC, RP, PN, CF, SF, AI, and PM: final approval of the article. All authors contributed to the article and approved the submitted version.

## Conflict of Interest

The authors declare that the research was conducted in the absence of any commercial or financial relationships that could be construed as a potential conflict of interest.
